# The CENP-T *C*-Terminus Is Exclusively Proximal to H3.1 and not to H3.2 or H3.3

**DOI:** 10.3390/ijms16035839

**Published:** 2015-03-12

**Authors:** Christian Abendroth, Antje Hofmeister, Sandra B. Hake, Paul K. Kamweru, Elke Miess, Carsten Dornblut, Isabell Küffner, Wen Deng, Heinrich Leonhardt, Sandra Orthaus, Christian Hoischen, Stephan Diekmann

**Affiliations:** 1 Molecular Biology, Fritz Lipmann Institute, Beutenbergstr. 11, D-07745 Jena, Germany; E-Mails: christian_abendroth@gmx.de (C.A.); hofi.antje@googlemail.com (A.H.); pkamweru@fli-leibniz.de (P.K.K.); elke.miess@med.uni-jena.de (E.M.); cdornblut@fli-leibniz.de (C.D.); isabell.kueffner@uni-jena.de (I.K.); hoischen@fli-leibniz.de (C.H.); 2 Department of Molecular Biology, Center for Integrated Protein Science Munich (CIPSM), Adolf-Butenandt-Institute, Ludwig-Maximilians-Universität Munich, Schillerstr. 44, D-80336 Munich, Germany; E-Mail: sandra.hake@med.uni-muenchen.de; 3 Department of Biology II, Center for Integrated Protein Science, Ludwig-Maximilians-Universität Munich, Planegg-Martinsried, D-82152 Munich, Germany; E-Mails: dengwen001@gmail.com (W.D.); h.leonhardt@lmu.de (H.L.); 4 PicoQuant GmbH, Kekulestr. 7, D-12489 Berlin, Germany; E-Mail: sandra.orthaus@googlemail.com

**Keywords:** centromere, kinetochore, Constitutive Centromere-Associated Network (CCAN), mitosis, histone variants, chromatin, Förster Resonance Energy Transfer (FRET)

## Abstract

The kinetochore proteins assemble onto centromeric chromatin and regulate DNA segregation during cell division. The inner kinetochore proteins bind centromeres while most outer kinetochore proteins assemble at centromeres during mitosis, connecting the complex to microtubules. The centromere–kinetochore complex contains specific nucleosomes and nucleosomal particles. CENP-A replaces canonical H3 in centromeric nucleosomes, defining centromeric chromatin. Next to CENP-A, the CCAN multi-protein complex settles which contains CENP-T/W/S/X. These four proteins are described to form a nucleosomal particle at centromeres. We had found the CENP-T *C*-terminus and the CENP-S termini next to histone H3.1 but not to CENP-A, suggesting that the Constitutive Centromere-Associated Network (CCAN) bridges a CENP-A- and a H3-containing nucleosome. Here, we show by *in vivo* FRET that this proximity between CENP-T and H3 is specific for H3.1 but neither for the H3.1 mutants H3.1^C96A^ and H3.1^C110A^ nor for H3.2 or H3.3. We also found CENP-M next to H3.1 but not to these H3.1 mutants. Consistently, we detected CENP-M next to CENP-S. These data elucidate the local molecular neighborhood of CCAN proteins next to a H3.1-containing centromeric nucleosome. They also indicate an exclusive position of H3.1 clearly distinct from H3.2, thus documenting a local, and potentially also functional, difference between H3.1 and H3.2.

## 1. Introduction

Chromosome segregation is executed by an evolutionary conserved molecular machinery which contains about 30 core subunits and recruits many additional regulatory proteins. A multi-protein complex, the “kinetochore”, assembles onto centromeric chromatin [1,2,3,4,5]. During mitosis, the kinetochore mediates the interaction between DNA and the mitotic spindle [6,7,8]. Kinetochores are built from an inner layer, directly contacting centromeric chromatin, and an outer layer, binding to the spindle microtubules. The inner kinetochore controls outer kinetochore assembly [9,10,11], influences microtubule binding [10,12], and contributes to epigenetic specification of centromeres [13,14,15,16].

Although centromeres are directly embedded in chromatin, specific DNA sequences are neither necessary nor sufficient for centromere function. Rather than centromeric DNA sequences, the centromere seems to be epigenetically defined, and its location inherited, by the H3 histone variant CENP-A, which replaces canonical H3 in centromeric nucleosomes [17,18,19,20,21,22,23,24,25,26,27,28]. CENP-A itself plays a role in propagating centromere identity as well as nucleating kinetochore formation [29,30]. CENP-A containing chromatin is sufficient for recruitment of the downstream centromere and kinetochore complexes [14,29,30,31,32,33,34]. CENP-A is stably transmitted at centromeres during mitotic [28,35,36,37] and meiotic [38] divisions, and its assembly is tightly cell cycle controlled [35,39,40,41]. CENP-A interacts with the histones H2A, H2B and H4 and, depending on cell cycle phase, is incorporated in octameric nucleosomes or tetrameric sub-nucleosomal particles [27,42,43,44,45]. This situation is under discussion [26]. In vertebrates, a group of at least 16 inner kinetochore proteins, called the Constitutive Centromere-Associated Network (CCAN), neighbors the CENP-A nucleosome [14,31,46,47,48,49,50,51,52]. The CCAN interacts with the outer kinetochore and contributes to maintain centromere identity by participating in CENP-A loading at every new cell division cycle [4,32,33,37,53,54,55,56,57,58].

CENP-C, conserved in all model organisms [17,55], recognizes and binds directly to the CENP-A nucleosome [33,51,59], but also to the CENP-N/L complex [60]. The CENP-C *N*-terminus binds the Mis12 complex [55,56], establishing a link to the outer kinetochore. Non-coding satellite RNA is also involved in centromere regulation [61,62,63,64,65,66,67]. Centromeric long non-coding RNA is described to be involved in Holliday Junction Recognition Protein (HJURP) targeting and subsequent CENP-A loading to the centromere [66].

The CCAN proteins can be grouped into several sub-complexes. First, the two-subunit CENP-N/L complex binds directly to CENP-A [26,32,60,68]. Second, the CCAN subunits CENP-H, CENP-I and CENP-K were proposed to form a complex [10,11,14] with CENP-M, classified in a distinct phenotypic class [14], recently being identified to stabilize this four-member complex [69]. CENP-I is required to generate a stable association of the RZZ complex (formed by Rod, ZW10 and Zwilch) and Mad1 with kinetochores and also inhibits their removal by dynein [70,71]. These four proteins were shown to be proximal to the CENP-N/L complex but also to the subunits of the CENP-T/W/X/S and CENP-O/P/Q/U complexes [14,31,48,50]. Third, the CENP-O/P/Q/U complex which interacts with CENP-R [72,73,74,75], has been implicated in microtubule binding and spindle checkpoint control [4]. Fourth, the CENP-T/W/S/X complex contains proteins with histone-fold domains that bind DNA and might form a nucleosome-like structure [37,51,76,77,78]. Together with CENP-C (see above), the CENP-T/W sub-complex contributes to outer kinetochore assembly: CENP-T/W interacts directly with the Ndc80 complex [8,54,55,56,79]. The CENP-S/X hetero-dimer is not essential for mitosis but plays a role in kinetochore stabilization [77,80]. Thus, the outer kinetochore is anchored to a multi-subunit binding platform within centromeric chromatin. An estimated 250 proteins comprise the full mitotic centromere-kinetochore complex [81], which ensures that this linkage is fully functional and error-free during cell division. Several of these interactions among CCAN subunits have been identified in *S. cerevisiae* and *S. pombe* [5,46,47,49,82,83,84,85,86,87,88,89] as well as in other organisms [90], suggesting a conserved plan of kinetochore assembly.

Interspersed with CENP-A- are histone H3.1- and H3.3-containing nucleosomes [91,92,93] (and probably also H3.2-containing nucleosomes which have not been explicitly studied), all of which have distinctive patterns of post-translational modification [94] within centromeres [95,96,97,98]. A third type of nucleoprotein particle was described comprising CENP-T, -W, -S and -X [76]. Thus, while CENP-A serves to identify and initiate kinetochore formation, the actual chromatin platform bound by kinetochore proteins seems to involve three different types of nucleosomes or nucleosome-like particles. CENP-N and the central domain of CENP-C directly bind the CENP-A nucleosome [32,59,68], kinetochore binding of CENP-T/W/S/X requires CENP-H/I/K/M [69]. The *C*-terminal domain of CENP-T was found in direct proximity to the *C*-terminus of H3 (using H3.1 as the labelled H3 variant [99]) but not CENP-A [77]. Thus, the inner kinetochore assembles as a multi-protein bridge between a CENP-A- and a H3-containing nucleosome [99]. In this structure, the path of the DNA remains unclear. Here, we asked if the identified proximity between the *C*-termini of CENP-T and H3 are H3 variant specific. Applying life cell microscopy [100], we found selectively H3.1 but neither H3.2, H3.3 nor mutated H3.1 proximal to CENP-T, CENP-W and CENP-M.

## 2. Results

Recently, we observed by acceptor-bleaching Förster Resonance Energy Transfer (FRET) that the *C*-terminus of CENP-T and both termini of CENP-S are in close proximity to one another and to the *C*-terminus of the histone H3 variant H3.1 [77,99]. The *N*-terminal end of CENP-S was found not to be close to the *C*-terminal end of the centromeric H3 variant CENP-A [77]. Here, we asked if CENP-T/W/S/X proteins are proximal to any of the H3 variants H3.1, H3.2 or H3.3, or if this proximity is H3 variant specific.

In order to obtain proximity data in the range of <10 nm—more than 20 times smaller than the Abbe microscope resolution—we carried out acceptor-bleaching FRET as described [68] since it is less error prone than other FRET techniques [100]. We measured FRET at many kinetochores in a number of different cells *in vivo* or *in situ*, using the FRET pair EGFP-mCherry [101]. Replacing this FRET pair by Clover-mRuby2 [102] improved donor stability and decreased data variance but did not generally improve our *in vivo* FRET results (data not shown). Cell synchronisation or specific cell cycle phase markers allowed us to select a particular time point in the cell cycle for our measurement, or to identify in the analysed cell when in the cell cycle our FRET measurement was carried out [27,73]. Our FRET measurements are based on a large body of control experiments [100,103,104].

Since histone H3 and CENP-T have long, flexible *N*-terminal arms, a fluorescent FRET tag at the *N*-terminus would be able to scan a large space so that positional local information can hardly be deduced [99]. Here, we therefore labelled the much shorter and less flexible *C*-terminal H3 and CENP-T arms.

**Figure 1:**
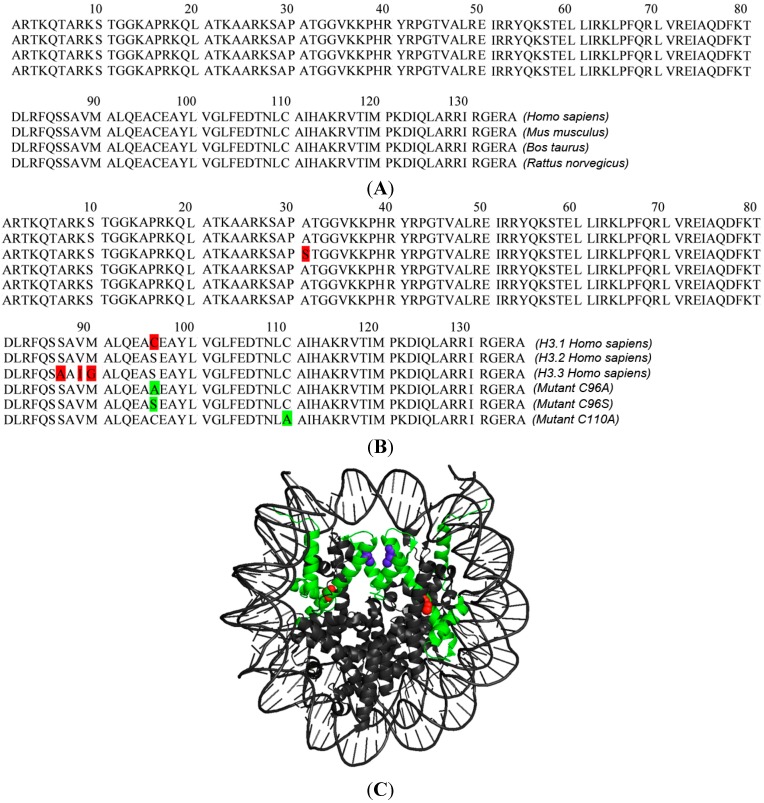
(**A**) H3.1 sequence conservation between *Homo sapiens*, *Mus musculus*, *Bos taurus* and *Rattus norvegicus*; (**B**) Sequences of the H3 variants H3.1, H3.2, H3.3 and the mutants constructed and analysed here. The mutant H3.1^C96S^ equals H3.2 (red: sequence deviations of H3.1 and H3.3 from H3.2; green: H3.1 mutants studied here); and (**C**) Nucleosome structure [105,106]. Both H3 copies are displayed in green with C96 in red and C110 in blue. C96 and C110 are located in different α-helices.

**Figure 2:**
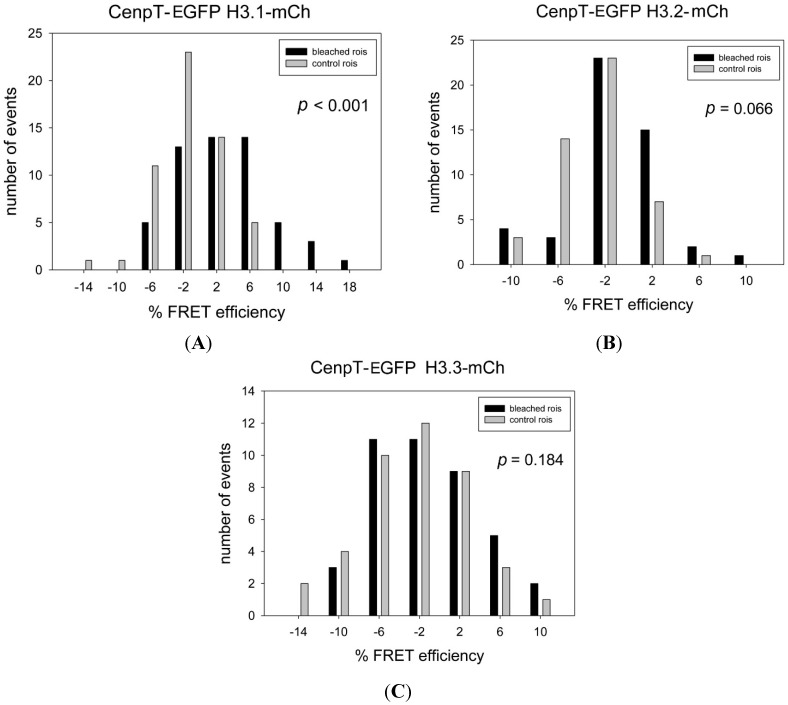
Acceptor-bleaching Förster Resonance Energy Transfer (FRET) between CENP-T-EGFP and (**A**) H3.1-mCherry; (**B**) H3.2-mCherry and (**C**) H3.3-mCherry. In one spot in the cell containing kinetochores, the acceptor mCherry is bleached and the donor EGFP fluorescence is measured before and after the bleach. From the difference, the FRET efficiency *E*_FRET_ is calculated and grouped according to 4% deviation categories (black bars). Parallel to this measurement, at another spot in the same cell also containing kinetochores, the acceptor is not bleached and the donor EGFP fluorescence is measured before and after the bleach. In this control measurement, the variation of the donor fluorescence is determined at a non-bleached cellular location. This donor fluorescence is treated in the same way resulting in *E*_VAR_ (grey bars). For every measurement, the *E*_FRET_ distribution was statistically compared to the control *E*_VAR_ distribution, obtaining the *p*-value.

The sequence of H3.1 is strongly conserved (Figure 1A). In humans it differs from that of H3.2 and H3.3 by only one and five amino acids, respectively (Figure 1B). First, we fused the H3 variants with the FRET acceptor mCherry, obtaining H3.1-mCherry, H3.2-mCherry and H3.3-mCherry. We repeated our FRET measurement between CENP-T-EGFP and H3.1-mCherry [99] and obtained a strong FRET result (*p* < 0.001, Figure 2A, Table 1), confirming our recent result and indicating that the *C*-termini of these proteins are very close to one another. Then, we measured acceptor-bleaching FRET in human HEp-2 cells between CENP-T-EGFP and either H3.2-mCherry or H3.3-mCherry. For both cases, we observed no FRET signal (*p* = 0.066 and *p* = 0.184, respectively; Figure 2B,C, Table 1). Thus, the proximity of the CENP-T *C*-terminus to the *C*-termini of the H3 variants is highly specific for H3.1. When deleting the *N*-terminal domain of CENP-T, no FRET signal was detected between CENP-T^ΔN^ (containing the amino acids 377–561) and H3.1-mCherry (*p* = 0.107, Table 1). We also measured FRET between CENP-T-EGFP and the three *N*-terminally tagged H3 variants mCherry-H3.1, mCherry-H3.2 and mCherry-H3.3. As expected, due to the long, flexible *N*-terminal arm of H3, we measured strong FRET (*p* < 0.001) in all three cases (data not shown). Thus, the two H3 variants H3.2 and H3.3 not showing FRET to CENP-T-EGFP when labelled at their *C*-termini were still found in some distal neighbourhood to the CENP-T *C*-terminus. H3.1 differs from H3.2 by the single amino acid C96, H3.2 equals H3.1^C96S^ (Figure 1B). Thus, our results indicate that C96 of H3.1 is essential for establishing close proximity to CENP-T.

**Figure 3:**
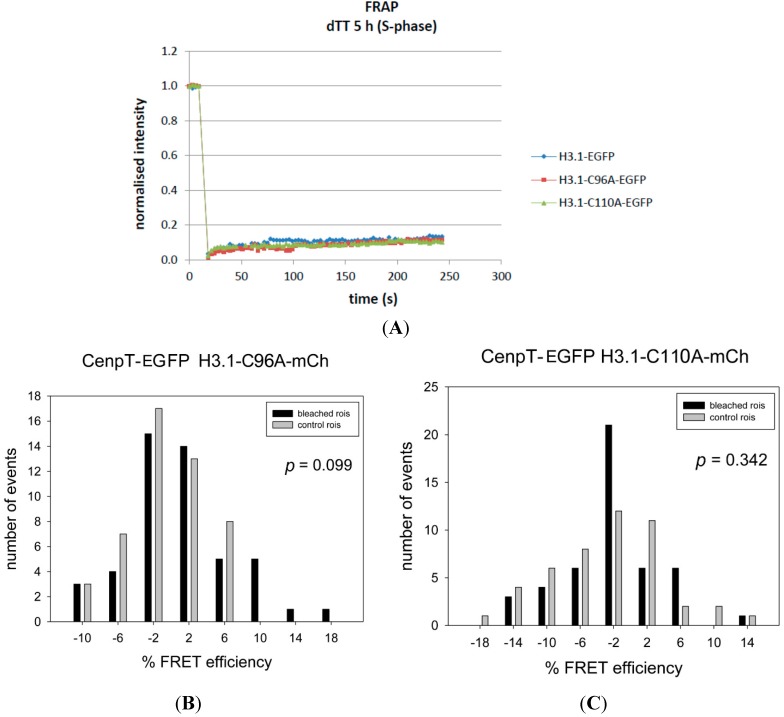
(**A**) Normalized Fluorescence Recovery After Photobleaching (FRAP) recovery curves of H3.1, H3.1^C96A^ and H3.1^C110A^ in S-phase (5 h after double thymidine block release) in transfected HeLa cells. All three proteins show the small and slow recovery typical for H3 [107]; (**B**,**C**) Acceptor-bleaching FRET between CENP-T-EGFP and (**B**) H3.1^C96A^-mCherry and (**C**) H3.1^C110A^-mCherry. The large *p*-values indicate the absence of a FRET signal.

Then, we constructed the mutant H3.1^C96A^ and fused it to mCherry, obtaining H3.1^C96A^-mCherry. First, we asked if this H3 mutant was indeed incorporated into centromeric chromatin. In transfected HeLa cells, we found GFP-H3.1^C96A^ incorporated into chromosomes as GFP-H3.1 (data not shown). Then, we measured the Fluorescence Recovery After Photobleaching (FRAP) of this H3 mutant in live HEp-2 cells and observed the same slow exchange behaviour as found for H3 [107] (Figure 3A). Furthermore, the H3 mutant localized at centromeres, together indicating the incorporation of these H3 mutant into centromeric nucleosomes. Then, we measured FRET between CENP-T and H3.1^C96A^ and found no FRET signal (*p* = 0.099; Figure 3B, Table 1). These results confirmed that C96 is essential for the close proximity between H3.1 and CENP-T.

**Table 1:** Summary of FRET measurements in this study between CCAN proteins and H3.1.

Donor Fusion	Acceptor Fusion	Mean *E*_FRET_ %	Mean *E*_VAR_%	Δ*E* %	*p*-Value	FRET	Number of Kinetochores Bleached (*N*_kin_)
CENP-T-EGFP *	H3.1-mCh *	3.03	−1.41	4.45	<0.001	++	55
CENP-T-EGFP	H3.2-mCh	−1.54	−2.89	1.35	0.066	−	48
CENP-T-EGFP	H3.3-mCh	−1.16	−2.66	1.50	0,184	−	41
CENP-T-EGFP	H3.1-C96A-mCh	0.83	−0.92	1.75	0.099	−	48
CENP-T-EGFP	H3.1-C110A-mCh	−1.82	−3.09	1.27	0.342	−	47
CENP-TΔN-EGFP	H3.1-mCh	3.53	1.97	1.56	0.107	−	43
EGFP-CENP-W	H3.1-mCh	2.00	0.27	1.73	0.251	−	36
EGFP-CENP-W	H3.2-mCh	2.05	1.85	0.20	0.801	−	85
EGFP-CENP-W	H3.3-mCh	0.49	1.51	−1.02	0.371	−	45
CENP-W-EGFP	mCh-H3.1	3.70	−2.10	5.80	<0.001	++	37
EGFP-CENP-W	CENP-T-mCh	7.79	0.19	7.6	<0.001	++	31
EGFP-CENP-W	CENP-B-mCh	4.37	0.75	3.60	<0.001	++	49
CENP-W-EGFP	mCh-CENP-B	5.07	0.67	4.40	<0.001	++	30
CENP-W-EGFP	CENP-B-mCh	1.81	0.79	1.02	0.200	−	42
EGFP-CENP-M	mCh-CENP-S	1.75	−0.22	1.97	0.064	−	32
EGFP-CENP-M	CENP-S-mCh	0.87	0.05	0.82	0.427	−	39
CENP-M-EGFP	mCh-CENP-S	4.00	−0.78	4.78	<0.001	++	38
CENP-M-EGFP	CENP-S-mCh	3.49	−0.42	3.91	<0.001	++	43
EGFP-CENP-M	H3.1-mCh	3.58	−1.42	4.99	<0.001	++	41
EGFP-CENP-M	H3.1-C96A-mCh	−0.75	−2.36	1,61	0.105	−	51
EGFP-CENP-M	H3.1-C110A-mCh	0.54	−1.49	2.03	0.011	−	53
CENP-M-EGFP	H3.1-mCh	2.91	−0.24	3.15	0.004	+	41
CENP-M-mCh	EGFP-CENP-U	6.08	−1.78	7.86	<0.001	++	54

*N*_kin_ is the number of analyzed kinetochores in a bleached area; *E*_FRET_ is the mean value of difference in donor-fluorescence after acceptor-bleach in a bleached region of the nucleus; *E*_VAR_ is the mean value of difference in donor fluorescence after acceptor-bleach in an unbleached region of the nucleus; Δ*E* = *E*_FRET_ − *E*_VAR_; *p*-Value obtained from *t*-test; A strong FRET signal is obtained when *p* < 0.001; ++, positive FRET; +, non-significant FRET; −, no FRET; * Data confirming results of [99]; In each FRET experiment, the number of unbleached control kinetochores was identical or very similar to the number of bleached kinetochores.

Next to C96, H3.1 contains a second cysteine, C110, in the α2 helix where both nucleosomal H3 proteins are closely apposed [105] (Figure 1C). Cysteine is one of the most rarely used amino acids in nature. It may be assigned to play a specialised functional role: cysteines can form disulfide bonds under oxidative conditions. Two C110 of the two H3 proteins within a single nucleosome might form an intra-nucleosomal disulfide bond [108], stabilising the H3-H4 tetramer. Two C96 of H3 proteins in neighbouring nucleosomes were speculated to form an inter-nucleosomal di-sulfide bond or a di-sulfide bond to a cysteine in a neighbouring protein [109]. Due to the nucleosomal H3 structure (Figure 1C) we consider it rather improbable that C96 and C110 form a *di*-sulfide bond within a single H3. In order to test if C110 is involved in establishing the proximity between CENP-T and H3.1, we constructed the H3.1 mutant C110A, fused it to mCherry, and measured FRET to CENP-T. Surprisingly, we did not observe FRET between CENP-T-EGFP and H3.1^C110A^-mCherry (*p* = 0.342, Figure 3C, Table 1). Thus, C110, although common in almost all H3 variants, is essential in H3.1 for establishing its proximity to CENP-T.

**Figure 4:**
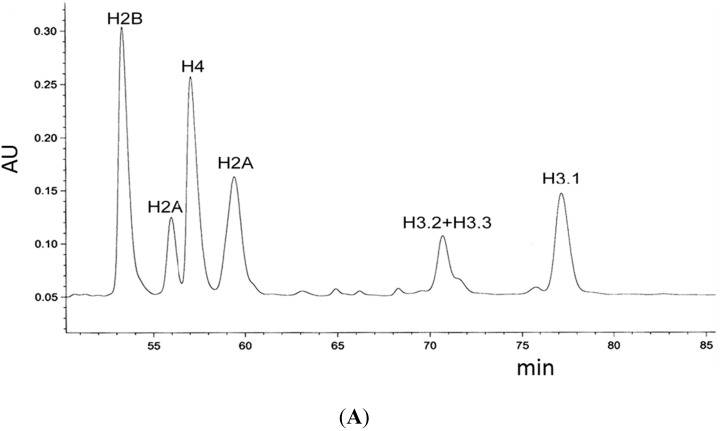

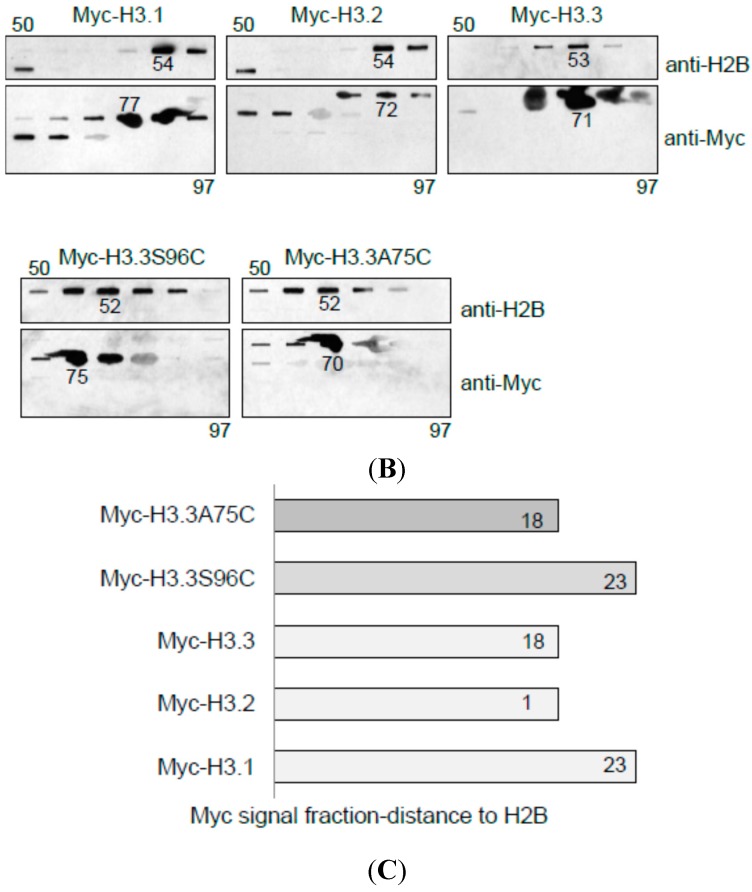
Analysis of myc-tagged H3 variant reverse-phase HPLC fractions. (**A**) Typical RP-HPLC profile of HeLa histones. Peaks that correspond to individual histones are labelled; (**B**) Slot immunoblots of RP-HPLC separated histone fractions 50–96 (from top left to bottom right) derived from HeLa cells transfected with indicated myc-tagged H3 wild type histones and mutants. Blots were stained with H2B (**top**) or myc (**bottom**) antibodies. H2B detection was chosen as reference elution point; (**C**) Graphical depiction of fraction numbers between strongest H2B and myc signal seen in (**B**).

C96 distinguishes H3.1 from H3.2 and, as we showed here, establishes the proximity to CENP-T. Thus, C96 must mediate properties to H3.1 that are detectable *in vivo*. We asked if we could identify such selective properties *in vitro*. We expressed the myc-tagged H3 variants H3.1, H3.2 and H3.3 in HeLa cells, acid-extracted and separated them in reverse phase HPLC (typical elution curve displayed in Figure 4A [94]. The HPLC fractions 50–96 were analysed by Slot-Blots and immuno-stained for H2B and the myc-tag (Figure 4B). We then determined the number of fractions between the strongest H2B and the strongest myc-tagged H3 band and found that similar to the endogenous proteins, myc-tagged H3.2 and H3.3 elute earlier than myc-H3.1 (Figure 4C). Then, we mutated H3.3, introducing a cysteine either at position S96 or at A75. Our analysis showed that the mutant H3.3^S96C^ eluted as H3.1 while mutant H3.3^A75C^ eluted as H3.3 (Figure 4C). Thus, the distinctive migration of H3.1 is critically dependent on the cysteine at position 96, a cysteine at position 75 did not result in a change of properties. These results indicate that C96 critically influences the molecular properties of H3 variants, in a way that H3.1 can be specifically recognised *in vivo*. In addition, we analysed the HPLC purified H3 fractions in gel electrophoresis and found that all three H3 variants showed monomers and dimers, however, H3.1 displayed additional bands suggesting the presence of oligomers (detectable up to octamers; data not shown). Dimer and oligomer formation would be consistent with di-sulfide bridges between one and two cysteines in the protein sequence, respectively.

We then analysed the proximity of H3 next to other CCAN kinetochore proteins. Earlier we detected the proximity between CENP-T, CENP-S and CENP-X *in vivo* [77], consistent with published results [76]. Both termini of CENP-S were found next to the H3.1 *C*-terminus [77]. CENP-W is the fourth member of the CENP-T/W/S/X complex [76]. We therefore labelled the *N*-terminus of CENP-W with EGFP and analysed its FRET neighbourhood to the *C*-terminus of CENP-T and the H3 variants. We found strong FRET between EGFP-CENP-W and CENP-T-mCherry (*p* < 0.001; Table 1), consistent with [76]. Surprisingly, however, EGFP-CENP-W did neither show FRET to H3.1-mCherry nor to H3.2-mCherry or H3.3-mCherry (Table 1). However, we did observe a FRET neighbourhood between the CENP-W *C*-terminus and the long flexible H3.1 *N*-terminus, both *in vivo* and *in situ* (Table 1). Thus, our data suggest that while both termini of CENP-S point towards H3.1, at least the CENP-W *N*-terminus points away from H3.1. We therefore searched for other kinetochore proteins being close to CENP-W and identified CENP-B. We observed strong FRET between EGFP-CENP-W and CENP-B-mCherry (*p* < 0.001) and between CENP-W-EGFP and mCherry-CENP-B (*p* < 0.001) but no FRET signal between CENP-W-EGFP and CENP-B-mCherry (*p* = 0.200, Table 1). This suggests that CENP-W and CENP-B have a well-ordered anti-parallel position next to one another within the kinetochore complex. Indeed, the *N*-terminus of CENP-W seems to point away from H3.1 towards CENP-B. Interestingly, by acceptor-bleaching FRET and by Fluorescence Lifetime Imaging (FLIM) we also found the *C*-terminus of CENP-B proximal to the *N*-terminus of CENP-I (data not shown).

**Figure 5:**
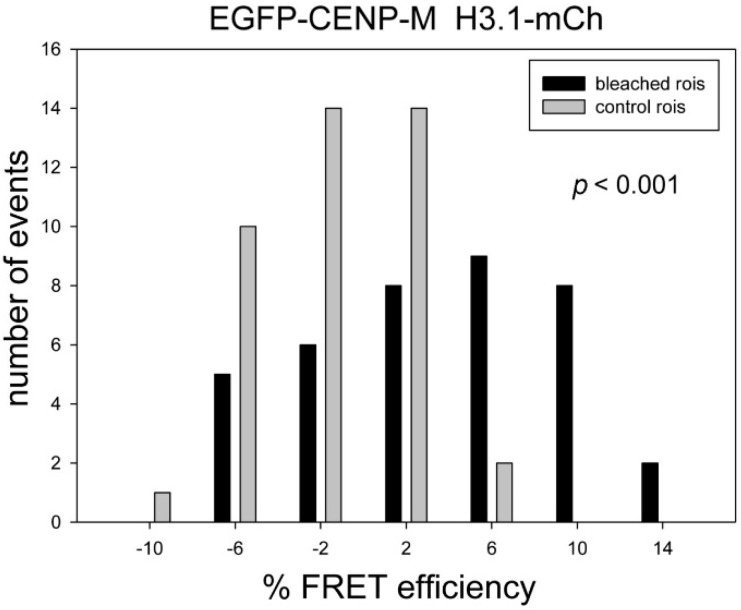
Acceptor-bleaching FRET between EGFP-CENP-M and H3.1-mCherry. The low *p*-value (*p* < 0.001) indicates a strong FRET signal.

Trying to identify protein neighbours of the CENP-T/W/S/X complex, we found by *in vivo* FRET that CENP-S is close to CENP-M. We detected strong FRET between the CENP-M *C*-terminus, but not its *N*-terminus, to both CENP-S termini (CENP-M-EGFP and CENP-S-mCherry: *p* < 0.001, CENP-M-EGFP and mCherry-CENP-S: *p* < 0.001, EGFP-CENP-M and CENP-S-mCherry: *p* = 0.427, EGFP-CENP-M and mCherry-CENP-S: *p* = 0.064, Table 1). This indicates that in the kinetochore CENP-M binding might be directional, with its *C*-terminus directed towards CENP-S. We speculated that when being close to CENP-S, CENP-M might also be proximal to H3.1. We thus analysed FRET between EGFP-CENP-M and H3.1 and observed strong FRET between EGFP-CENP-M and H3.1-mCherry (*p* < 0.001, Figure 5, Table 1). As for CENP-T, we asked if this proximity is H3.1 specific. We measured FRET between EGFP-CENP-M and the H3.1 mutant fusion proteins H3.1^C96A^-mCherry and found no FRET signal (H3.1^C96A^: *p* = 0.105; Table 1). Analysing EGFP-CENP-M and the H3.1 mutant H3.1^C110A^-mCherry resulted in a strongly reduced FRET signal (*p* = 0.011; Table 1). Thus, also the proximity of the CENP-M *N*-terminus to H3.1 is strictly dependent on the presence of C96. For the *C*-terminus of CENP-M, we observed a smaller FRET signal (CENP-M-EGFP to H3.1-mCherry resulted in *p* = 0.004; Table 1), supporting a directional incorporation of CENP-M into the kinetochore complex. By yeast-two-hybrid experiments, we searched for other CCAN proteins which might interact with CENP-M and identified a weak signal for CENP-U. Therefore, by *in vivo* FRET we studied the neighbourhood of CENP-M and CENP-U. We observed strong FRET between CENP-M-mCherry and EGFP-CENP-U (*p* < 0.001; Table 1). We then asked if, in the human cell, this proximity between CENP-M and CENP-U results from a direct pairwise interaction between these two proteins. By mammalian-three-hybrid (F3H, see Experimental Section), we could not detect a direct strong pairwise interaction between CENP-M and CENP-U, nor with any other CCAN protein (data not shown). By Fluorescence Cross Correlation Spectroscopy in the nucleoplasm, however, we identified an interaction between CENP-M and CENP-I, confirming published data [69]. Earlier we had detected FRET between Cerulean-CENP-I and EYFP-CENP-U [52]. Thus, we find that both proteins, CENP-M and CENP-I, are proximal to the *N*-terminus of CENP-U.

By *in vivo* FRET in human cells, we thus identified the local proximity of CENP-T, CENP-S and CENP-M next to specifically H3.1 in the interphase kinetochore.

## 3. Discussion

The inner kinetochore multi-protein complex assembles at the centromere. Proximity and protein–protein interaction data suggested that the complex bridges two nucleosomes, one containing the H3 variant CENP-A, the other H3.1 [99]. Recently, we showed that the CENP-T/W/S/X complex is close to the H3.1 nucleosome [77,99]. Here, we showed by *in vivo* FRET that this proximity is selectively established for H3.1 but not for H3.2 and H3.3. Furthermore, this proximity was also not detected when mutating the amino acid Cys96 which distinguishes H3.1 from H3.2, to Ala96 or Asp96. Interestingly, also when mutating Cys110 of H3.1 to Ala110, this proximity was lost. Thus, H3.1 is incorporated into a nucleosome having a particular location at the centromere, and neither H3.2 nor H3.3 was detected at this place. We observed a clear distinction between H3.1 and H3.2/H3.3, which at the centromere may be functional.

Since the proximity between the *C*-termini of CENP-T and H3 was detected only for H3.1 but not for H3.2, Cys96 must be specifically recognized over Ser96. The trimeric Chromatin Associated Factor-1 (CAF-1) [110,111] is not able to distinguish these two amino acids since it associates with both H3.1 and H3.2, and mediates their DNA synthesis-coupled deposition [112]. We speculated that the long, flexible *N*-terminal arm of CENP-T might contain a specific H3.1 recognition domain. However, preliminary experiments with CENP-T mutants were not fully supportive. Alternatively, centromere binding RNA could be involved in H3.1 recognition [66,67]. Here, we show that a cysteine at position 96 results in specific molecular properties which make H3.1 distinguishable by HPLC from H3.2. When a cysteine is introduced into H3.3 at position 96, now also H3.3^S96C^, but not H3.3^A75C^, elutes as H3.1. Thus, C96 introduces specific molecular properties into H3.1. It remains unclear, however, how this difference is recognized *in vivo*. Our *in vitro* results are consistent with C96 and C110 being involved in di-sulfide bonds between isolated proteins. C110 of two H3 might form intra-nucleosomal *di*-sulfide bridges [108,113] stabilizing nucleosome structure. However, it remains unclear if C96 forms *di*-sulfide bridges *in vivo*, either within the same or between neighboring nucleosomes, or to proximal proteins [109].

Although differing only by C96, H3.1 and H3.2 carry significantly different post-translational modifications [94]. The H3.2 marks have been associated with gene silencing (K27 *di*- and trimethylation) and the formation of facultative chromatin while the H3.1 modifications are linked to gene silencing (K9 dimethylation) as well as to gene activation (K14 acetylation), suggesting that both human H3 variants may have separate biological functions [94,109]. Potentially, also C96 (or, alternatively, S96 in the other H3 variants) might be specifically post-translationally modified. *In vivo*, C110 was found glutathionylated in H3 variants; however, such a modification was not found for C96 in H3.1 [114]. Taken together, the molecular basis for the specific recognition of H3.1 over H3.2 *in vivo* remains unresolved.

The larger variation of H3 variants in humans is not conserved: *S. cerevisiae* and *S. pombe* contain only one type of H3 (H3.3 and H3.3 like, respectively) while *Arabidopsis*, *Xenopus* and *Drosophila* contain two types (H3.2 and H3.3). Thus, the local centromeric structure observed here is not of a general nature and may even be unique to humans. Probably, organisms not having H3.1 have a more or less modified centromeric structure next to CENP-T/W/S/X. Potentially, a missing H3.1 comes with a CCAN assembly modified in structure and/or composition. In general, kinetochores seem to be similar in function although they differ in molecular details.

In our experiments, a FRET signal appears when the distance between the donor and acceptor fluorophore becomes smaller than about 10 nm (to become detectable in the cell, probably smaller than 8 nm [100]). Due to the dimensions of the β-barrels around the EGFP and mCherry fluorophores, for sterical reasons the smallest distance between the fluorophores is about 4 nm. Thus, to allow for FRET, donor and acceptor must be in the direct molecular neighborhood. Via a short peptide linker, donor and acceptor were fused to kinetochore and histone proteins. According to fusion protein and linker dimensions, the distance between these fused proteins might be larger than that of donor and acceptor. In particular in a stable multi-protein complex, a FRET signal between donor and acceptor does not necessarily indicate a direct interaction between the fused proteins. Furthermore, the bulky fluorescent tags might influence the local complex architecture. Thus, a detailed structural interpretation of *in vivo* FRET data is limited. Nevertheless, our FRET proximity analysis confines the relative positioning of some inner kinetochore proteins next to a H3.1-containing nucleosome. We had shown that not only the *C*-terminal domain of CENP-T but also both termini of CENP-S are close to the *C*-terminus of H3.1 [77,99]. Here, we found that the *N*-terminal domain of CENP-W, another member of the CENP-T/W/S/X complex, seems to point away from the H3.1 *C*-terminus and is close to the CENP-B *C*-terminus while the CENP-B *N*-terminal domain is close to the CENP-W *C*-terminus. CENP-B binds to the CENP-B box located on every other α-satellite centromeric DNA [104,115,116,117]. Our results position CENP-B next to the CENP-T/W/S/X complex. CENP-B thus binds to centromeric linker DNA either directly next to CENP-A nucleosomes and separately also directly next to CENP-T/W/S/X nucleosomal particles [78], or to a linker bridging these two structures. We also detected CENP-M next to CENP-S and observed that the CENP-M *N*-terminus is also proximal to the H3.1 *C*-terminus. Our FRET studies showed that, again, this proximity is H3.1 specific and is not established for the H3.1 mutants H3.1^C96A^ or H3.1^C110A^. Thus, CENP-M is in direct proximity to the CENP-T/W/S/X complex and the centromeric nucleosome selectively harboring H3.1. Furthermore, we found that the CENP-M *C*-terminus is proximal to the CENP-U *N*-terminus. Our FRET analyses place CENP-M, and thus probably the CENP-M/H/I/K complex [69], in direct proximity to the H3.1 containing nucleosome and the CENP-T/W/S/X complex on one side and the CENP-P/O/R/Q/U complex on the other side. It remains open if CENP-M is able to directly bind to CENP-U: we detected a weak two-hybrid signal in yeast but not in human cells.

**Figure 6:**
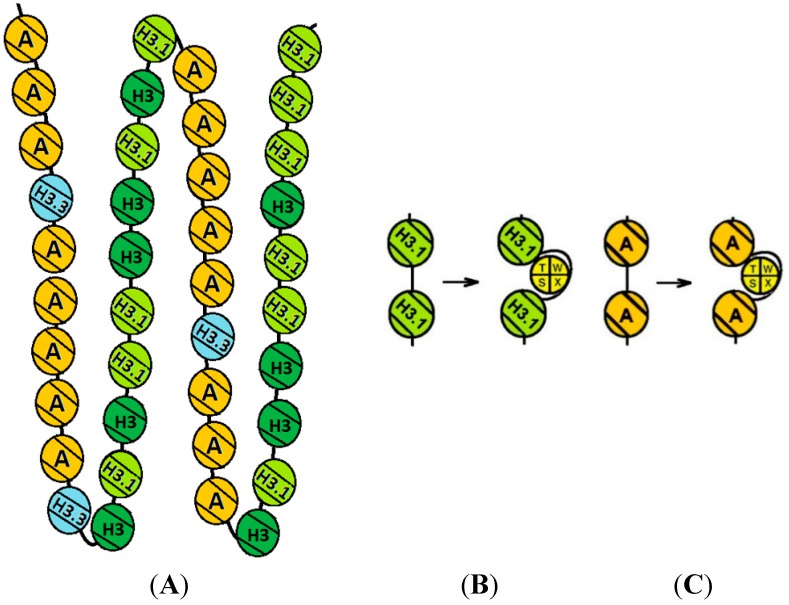
Boustrophedon kinetochore structure [96]. (**A**) One loop of a potential centromeric chromatin structure at late S phase. CENP-A dominates one stretch of nucleosomes while H3 variants are distributed on the folded-back strand. The CCAN complex is supposed to fold between a CENP-A on the one and H3.1 on the other strand [99]; (**B**,**C**) CENP-T/W/S/X may be wrapped around by DNA in cis to CENP-A-containing (**C**) or H3.1-containing nucleosomes (**B**).

We consider the path of the DNA in the centromeric complex still unresolved: either, the three nucleosomal particles (containing either CENP-A, H3 or CENP-T/W/S/X) lie in cis along the chromosomal DNA strand next to one another, or alternatively, proximity is established by folding of the chromatin fiber back onto itself, bringing distant regions with respect to DNA into contact in trans (Figure 6A). Centromeric chromatin was suggested to fold into a boustrophedon structure with 10 nm strands folding back onto themselves in a three-dimensional structure [96]. When one 10 nm strand contains CENP-A and the folded back structure H3.1 nucleosomes (Figure 6), the CCAN kinetochore complex might bridge these two strands with CENP-N on the one end (next to CENP-A) and parts of the CENP-T/W/S/X complex and CENP-M on the other end (next to H3.1). When centromeric DNA wraps around the CENP-T/W/S/X complex [78], in centromeric chromatin it remains unclear if this DNA is in cis to CENP-A- or to H3.1-containing nucleosomes. Recently, a CCAN recruitment pathway has been suggested with CENP-C, probably together with CENP-N/L binding to CENP-A, being required for CENP-H/I/K/M complex, and CENP-H/I/K/M required for CENP-T/W recruitment [69]. If the centromeric DNA path would follow this kinetochore assembly pathway and wrap the CENP-T/W/S/X complex *in vivo*, CENP-A nucleosomes and CENP-T/W/S/X nucleosomal particles would be in cis (Figure 6C), with CENP-B and linker Histone H1.0 [104] probably binding the linker between them. On the other hand, although in unfolded centromeric short fiber chromatin, CENP-T, CENP-H and CENP-C co-localised with CENP-A, in more extended fibers of CENP-T, now different from CENP-H and CENP-C, located at interspersed chromatin regions between CENP-A domains [96]. The reason for this observation might potentially be that linker DNA connecting two H3.1 nucleosomes wraps around the CENP-T/W/S/X complex (cis to H3.1 and trans to CENP-A, Figure 6B). Please note that also this DNA contains CENP-B boxes. Experiments are underway to resolve the path of centromeric DNA in CENP-A containing chromatin.

## 4. Experimental Section

### 4.1. Cell Culture

HeLa, HEp-2 and U2OS (obtained from ATCC, Manassas, VA, USA) cells were cultured in DMEM with 10% FCS as previously described [68,118]. Cell synchrony using the double thymidine protocol was as described by [37].

### 4.2. Plasmids

Cloning of CENP-T and CENP-U has been described elsewhere [52,77]. Plasmid pDF149, encoding the LAP-CENP-M fusion protein, was a kind gift of Dan Foltz and Iain Cheeseman. CENP-W was obtained directly by PCR from pCMV-Sport (imaGenes, Berlin, Germany) containing the gene of C6orf173 (CENP-W). Full length coding sequences were amplified by PCR (Expand high fidelity^PLUS^ PCR System; Roche, Penzberg, Germany) using primers incorporating flanking attB recombination sites and transferred into vector pDONR221 by BP recombination reaction (Invitrogen, Carlsbad, CA, USA). Genes were then transferred by LR recombination reactions into modified pFP-C and pFP-N (BD Biosciences, Clontech, Palo Alto, CA, USA) based Destination vectors. Vectors pEYFP-N1-H3.1/2/3 harbouring the DNA-sequences coding for H3.1-EYFP, H3.2-EYFP, and H3.3-EYFP, respectively, were based on the expression vector pEYFP-N1 (Clontech) and are described in [119]. pmCherry-N1-H3.1/2/3 coding for H3-mCherry fusion proteins resulted from the replacement of EYFP with mCherry in AgeI-BsrGI digested pEYFP vectors. For construction of pmCherry-C3-H3.1 containing the gene for mCherry-H3.1, pmCherry-N1-H3.1 was digested with HindIII and BamHI and the 455-bp H3.1 fragment was ligated into HindIII and BamHI opened pmCherry-C3. The genes of H3.1 variations containing exchanges of amino acids C96 to A96 and C110 to A110 were amplified by PCR using primers introducing the relevant mutations and incorporating flanking attB recombination sites and transferred into vector pDONR221. Genes were then transferred by LR recombination reactions into destination vectors. Plasmid pH-G-CenpT was used for amplification and subsequent GATEWAY-cloning of a CENP-T deletion mutant (CENP-T-ΔN) carrying the histone fold of CENP-T and lacking aas 1–376 (start with A377) by PCR (Expand high fidelity^PLUS^ PCR System) applying forward primer 5'-GGGGACAAGTTTGTACAAAAAAGCAGGCTTCGAAAACCTGTATTTTCAGGGCGCCACCATGGCTGAGGCTGACGGGCCAG-3' and reverse primer 5'-GGGGACCACTTTGTACAAGAAAGCTGGGTCTGGGCAGGGAAGACAGAGTT-3'. All constructs were verified by DNA sequencing (MWG Biotech, Ebersberg, München, Germany). Myc-tagged H3 variant plasmids were generated by PCR amplification of H3 variant cDNAs and cloning into pCMV-Myc plasmid (Clontech) via EcoRI/NotI digestion sites. Point mutants were introduced based on the site-directed Quickchange mutagenesis protocol by Stratagene (La Jolla, CA, USA).

### 4.3. Cell Lines and Transfection

For FRET and FRAP experiments, appropriate constructs were transfected into HeLa, HEp-2 and U2OS cells by electroporation and assayed after 24–48 h. Transfection of HeLa cells with pCMV-Myc-H3 plasmids was performed with Lipofectamine (Invitrogen) according to the manufacturer’s instructions.

### 4.4. F3H

BHK cells (obtained from David Spector) containing a *lac* operator repeat array [120] were cultured in DMEM medium with 10% FCS (Fetal Calf Serum) and seeded on coverslips in 6-well plates for microscopy. After attachment, cells were co-transfected with expression vectors for the indicated fluorescent fusion proteins and a LacI-GBP fusion [121,122] using polyethylenimine (Sigma, St. Louis, MO, USA). After about 16 h, cells were fixed with 3.7% formaldehyde in PBS for 10 min, washed with PBST (PBS with 0.02% Tween), stained with DAPI and mounted in Vectashield medium (Vector Laboratories, Servison, Switzerland). Samples were analyzed with a confocal fluorescence microscope (TCS SP5, Leica, Wetzlar, Germany) equipped with a 63 × 1.4 numerical aperture Plan-Apochromat oil immersion objective as described [122].

### 4.5. Acceptor Photobleaching Based FRET Measurements (AB-FRET)

FRET experiments were conducted as described previously [68,73,77,100,103,104]. EGFP fluorescence before and after mCherry-bleaching of a 4–10 µm^2^ region of interest (ROI) that contained 2–5 centromeres was compared resulting in FRET efficiency (*E*_FRET_) values. Additionally, EGFP fluorescence in an unbleached area that contained an equal amount of centromeres (±1) was analysed resulting in control FRET efficiency (*E*_VAR_). The determined *E*_FRET_ and *E*_VAR_ values were classified into 4% deviating categories and the number of counts for each category is shown in a bar diagram. Both input groups (*E*_FRET_ and *E*_VAR_ values) were statistically evaluated using Mann-Whitney Rank Sum test.

### 4.6. Fluorescence Recovery after Photobleaching (FRAP)

Fluorescence Recovery After Photobleaching (FRAP) experiments were carried out on a Zeiss LSM 510 Meta confocal microscope (Carl Zeiss, Jena, Germany) using a C-Apochromat infinity-corrected 1.2 NA 63× water objective and the 488 nm laser line for GFP, essentially as described previously [37,68,100]. For the FRAP measurements, the indicated fluorescent protein fusion proteins were transfected into HeLa cells. Five or 10 images were taken before the bleach pulse and 50–200 images after bleaching H3 fusion proteins at ROIs containing centromeres with an image acquisition frequency of 0.5–1.0 frames per second at 1% laser transmission to avoid additional bleaching. Relative fluorescence intensities were quantified as described [123,124] using Excel (Microsoft, Redmond, WA, USA) and Origin software (OriginLab, Northhampton, MA, USA).

### 4.7. Analysis of Myc-Tagged H3 Variants by Reverse-Phase HPLC and Fraction Analysis

Acid-extraction of histones from HeLa cells expressing myc-tagged H3 variants was done as described in [125] and RP-HPLC purification of histones was performed as mentioned in [94]. Collected fractions were dried under vacuum, resuspended in water and a part analyzed by Slot Immunoblotting. Antibodies used were: anti-H2B (Upstate/Millipore, Billerica, MA, USA) and anti-Myc (clone 9E10, Sigma).
